# The Need for Evidence-Based Interventions to Reduce Food Insecurity Among College Students

**DOI:** 10.13023/jah.0202.01

**Published:** 2020-04-15

**Authors:** Stephanie Jilcott Pitts, Sarah Brothers

**Affiliations:** Brody School of Medicine, East Carolina University; Department of Sociology, Yale University

**Keywords:** Appalachia, food insecurity, college students, food resources, intervention

## Abstract

Food insecurity is defined as insufficient resources to meet food needs. This is a global problem but is not confined to those countries identified as poor. One group of Americans who are particularly affected, and for whom the effects are particularly severe, is college students.

Food insecurity, defined as insufficient resources to meet food needs,^1^ is predominantly discussed as a problem in poor countries outside the United States. Food insecurity is indeed a global problem. In 2018, 26.4% of the world’s population, over 2 billion people, suffered from food insecurity.^2^ However, food insecurity is also a major problem for Americans. In 2018, 11.1% of Americans, or 37.2 million people, were food insecure.^1^

One group of Americans who are particularly affected by food insecurity, and for whom the effects are particularly severe, is college students. Food insecurity rates are much higher among college students than the general population. Approximately one-third to two-thirds of college students are marginally food insecure.^3^ In a recent survey, 49% of college students reported that they worried they would run out of food before they had funds to purchase more, and 18% reported that they lost weight because they lacked money to buy food.^4^ Food insecurity among college students is not limited to those from poverty; high rates are found among students from diverse socioeconomic backgrounds throughout the U.S.^5^

Food insecurity for college students impacts not only educational attainment and acquisition of job skills, it impacts physical and mental health. It is associated with lower grade point averages,^6^,^7^ poorer physical health,^6^ and higher rates of mental health issues including depression and anxiety.^3^,^8^ In addition, undernutrition between the ages of 18 to 24, the age of most college students, affects skeletal growth and neurodevelopment and can lead to long-term health problems including osteoporosis and obesity.^9^,^10^

During this developmental stage, reward-seeking areas of the brain are dominant, while areas responsible for planning and self-control are still forming.^11^ Thus, college students may need more support than adults to budget, plan, purchase, and prepare nutritious meals. Eating habits and food preferences established during these years will persist into adulthood, and if these habits are unhealthy, they can lead to long-term health problems.^12^

Despite the prevalence, severity, and long-term impact of food insecurity for college students, food insecurity among college students is sparsely examined.^3^,^13^ Current solutions do not fully remedy food insecurity. Although food insecurity is so much higher among college students than the general population, only 6.4% of students receive Supplemental Nutrition Assistance Program (SNAP) benefits.^14^ Studies find that over 60% of college students who receive SNAP benefits and 43% of those who have a school meal plan are still food insecure.^14^,^15^ The problem of food insecurity among college students will likely be exacerbated given the current COVID-19 pandemic. Many students did not have enough food while on campus; this problem could worsen now that many campuses are closed.

Three papers in this issue of the *Journal of Appalachian Health* demonstrate that food insecurity severely affects college students in Appalachia.^16^–^18^ One paper^16^ found that food insecurity affected almost half of the participants, yet only 17% of the food insecure students were pantry shoppers. Many food-insecure students said that they did not use the pantry because they felt embarrassed asking for help (21%) or felt others needed it more (30%). Nevertheless, the majority of those who used the pantry said it enabled them to spend more on other necessities (56%) and some said their job performance improved (18%).

However, as another paper demonstrates,^17^ available food pantries sometimes do not provide the level of healthy foods that students need. Interventions to improve the healthfulness of food pantries on college campuses, as done in pantries elsewhere,^19^ could lead to improved health among pantry shoppers. The third paper^18^ in this issue examined food insecurity among students with diagnosed medical disorders. The authors found that almost two-thirds of students with diagnosed medical disorders were very food insecure, perhaps in part because they spent more money on medications and medical devices than students without medical disorders. This study also found that academic performance declined as food insecurity increased, particularly for students with medical disorders. These papers illustrate that more work needs to be done to implement and evaluate effective interventions to reduce food insecurity among college students throughout the U.S.

Some creative solutions have already been implemented. For instance, recently Jon Bon Jovi opened a donation-based, pay-what-you-can restaurant that encourages people to volunteer in exchange for their meal on a New Jersey college campus in order to combat hunger among college students. Both California and New Jersey have state grants to help public colleges provide food for their students. These solutions should be examined for their effectiveness at reducing the prevalence of food insecurity. If they are effective, more states should follow their lead.

Research on food insecurity among college students reveals that many do not have the food they need while they are still developing intellectually, emotionally, and physically. The transition to college itself, exacerbated by lack of financial management skills, may increase food insecurity.^5^ Studies highlight a wide range in severity of food insecurity, and some have postulated the need for validated food security instruments that more accurately measure food insecurity in the college population.^3^ Different levels of intervention may be necessary. For example, for those worried about running out of food before they have money to purchase more, budgeting and cooking skills may be needed. Innovations in this area could include smart phone mobile applications to assist students when food shopping, and text message interventions to help students make wise budgeting choices. For the students who say they lost weight because they lacked money to buy food, interventions might include more tangible resources and support, such as expanded use of food pantries, additional free dining hall meal swipes, and increased financial support. Understanding students’ perspectives on food insecurity is critical to developing effective interventions. Additional qualitative and quantitative research is needed to ensure that interventions increase food security and ultimately improve their overall health.

Research-tested, evidence-based interventions are necessary to allow all students to maximize their education, and put their energy into establishing a stable, successful future. In addition to helping them focus on their education, alleviating food insecurity during this critical time period will help them be healthy and productive members of society for the rest of their lives.
